# Case of Dr. J. K. Rodgers

**Published:** 1852-02

**Authors:** 


THE CASE OF DR. J. K. RODGRRS.

We publish the case of the late Dr. Rodgers without the slightest reference to its controversial bearing. We have no intention of taking any part in the unpleasant controversy; but from the distinction of the deceased as a surgeon, and the intrinsic interest of his case, we supposed a history of it would be acceptable to our readers. The gentlemen who appear in the case are among the most eminent physicians of whom New York can boast, and a difference of opinion among such men only shows how difficult and uncertain is diagnosis in cases of deep-seated disease. We are sure that those who are the most deeply read, and of the largest experience in medicine, will be the slowest to deem uncharitably in such circumstances. In this case, Dr. Hosack appears to have discovered the nature of the disease from the beginning, and his colleagues would seem equally to have mistaken it; but in the next case, the result might be a change in the relative position of the parties; for superior skill in a single instance is by no means proof of general superiority. But conceding the accuracy of Dr. H's diagnosis, we think he was far from being justified in giving so confident a prognosis. We much doubt whether the treatment he proposed would have arrested the disease of the liver disclosed by the autopsy, though it cannot be denied that it was a plan that promised well in the premises. The case is an instructive one and will be read with interest.

We subjoin the following obituary notice of Dr. Rodgers, which we find in the *New York Journal of Medicine,* for January:

"He was born in 1793, and was the son of Dr. J. R. B. Rodgers, a practitioner in this city, of great eminence. He was educated at Princeton, N. J., and studied medicine in this city, under the late Prof. Wright Post, of the College of Physicians and Surgeons. He graduated in 1816, and was at one time House Surgeon to the New-York Hospital. Soon after the completion of his professional studies, he went to Europe, where he spent two years, either in Paris or London, principally in the latter, where he attended the lectures of Sir Astley Cooper, Mr. Benjamin Brodie, Travers, Abernethy, &c., who soon discovered in him the evidences of great promise, and extended to him those marks of attention, which gave evidence of their estimate of his character and ability. Two years after his return to this country, viz., 1820, he, in connection with Dr. Delafield, established the New York Eye and Ear Infirmary. In 1822 he was appointed one of the Surgeons of the New York Hospital, a post which he continued to fill, with distinguished ability, up to the time of his death. Dr. Rodgers was, in an eminent degree, practical and truthful in all his habits, and to these traits may we attribute his great abhorrence to quackery, in all its forms. As an operator, he was alike distinguished for his ease and adroitness, and as a man for his sterling integrity and noble and generous mind. He never disguised the truth for the *eclat* of an operation. His principal operation, one which will do him honor throughout the surgical world, and all time, was the ligation of the left subclavian artery, within the scaleni muscles, in 1846, and recorded in the 7th volume of this Journal. His contributions to our periodical literature were not numerous, but yet sufficient to leave a mark which will not be easily erased. His death is deeply deplored by us all. The New York Academy of Medicine, of which he was one of the Vice Presidents, the New York County Medical Society, and the Trustees and Students of the College of Physicians and Surgeons, passed appropriate resolutions on the occasion of his decease."

